# Hospital and Institutional News

**Published:** 1916-03-25

**Authors:** 


### HOSPITAL AND INSTITUTIONAL NEWS.

### MACMILLAN AND CO., LTD., AND ANOTHER V. THE NURSING PRESS, LTD., AND OTHERS.

With reference to the reports of this case from the *Times* and the summing-up of the Judge, Mr. Justice Ridley, which we published in The Hospital of 18th inst., pages 553 to 556, many readers may be interested to know that a full and accurate report of the whole proceedings will be issued in pamphlet form shortly by the publishers of The Hospital, The Scientific Press, Ltd., 28 and 29 Southampton Street, Strand, London, W.C.

### "ANOTHER BROWNLOW HILL INFIRMARY SCANDAL."

The Scientific Press, Limited, have received from the Clerk to the Select Vestry of the Parish of Liverpool a letter calling for immediate justification for the publication of the paragraph which appeared in our issue of March 18, headed "Another Brownlow Hill Infirmary Scandal" (see page 537), and giving three quotations from that paragraph. The justification is the report of the Vestry's Visiting Surgeon, Dr. Evans, which was discussed at their meeting on March 7, and the observations thereon of some members of the Vestry as reported in the *Liverpool Evening Express* of that date. We have complied with the further request of the Vestry Clerk by sending him the name of the firm of solicitors which will accept service of what he refers to as "legal notice," if the Vestry should think it well to submit Dr. Evans' report and our paragraph to investigation in a court of law.

### LORD KNUTSFORD AT HOME AGAIN.

Lord Knutsford has been moved to-day to his house in Eaton Place. It is good to hear as we go to press that he stood the journey very well. We may, therefore, hope that his recovery will now be rapid and complete.

### THE DIRECTOR-GENERAL OF THE A.M.S.

A curious rumour arose last week concerning the Director-Generalship of the Army Medical Service to the effect that Sir A. Keogh was resigning that important post. This rumour has been officially denied, and the same *communiqué* announces that Surgeon-General Babtie, V.C., is to act as his assistant at the War Office. In the semi-official eulogy of Sir A. Keogh's work since his appointment a well-deserved tribute is paid to his energy and organising capacity. Sir Alfred, like Lord Kitchener, has a great personality and is a tireless worker; but, like the Secretary for War, he has suffered by being charged with the control of too many details, and the appointment of Surgeon-General Babtie is evidence that this fact has at last penetrated the official mind. The recital of the splendid work of organisation which Sir Alfred has performed in remedying the deficiencies which faced him on accepting office is convincing, and he is one of the pleasantest of co-workers. The official defence of Sir A. Sloggett against the attacks of Mr. McNeill in the House of Commons seemed but half-hearted. Indeed, Mr. McNeill has received much support in the Press, and even more in the private conversations of those who know most—namely, the officers of the Royal Army Medical Corps.

### THE CONSCIENTIOUS OBJECTOR IN MILITARY HOSPITALS.

On the day we go to press Mr. Newdegate is to ask the Under-Secretary for War whether it is true that 200 orderlies have been transferred from a military hospital to undertake duties elsewhere, and that 200 conscientious objectors of military age have been appointed to take their places. The same member also wants to know whether the policy of the Government is to employ conscientious objectors as orderlies at military hospitals. The answers to these questions will be public property by the time this issue is in the hands of our readers. We can in the meanwhile only hope that the allegation in the first question is untrue, and that an equally satisfactory answer will be forthcoming to the second question. The country believes, and probably with justice, that about 90 per cent. of the conscientious objectors are merely scrim-shankers and cowards; and to provide these obnoxious people with safe and comfortable billets in hospitals will inevitably lead to trouble between them and the wounded patients. The conscientious objector, real or simulated, should be provided with the hardest and messiest sort of fatigue work that can be found for him. He who will not serve his country when he could deserves no consideration from those who bear his burdens for him; and we should be glad to see him disfranchised for life.

### THE PENSIONS OF TUBERCULOUS EX-SOLDIERS.

On behalf of soldier patients in sanatoria who are said to be in difficulties about pensions, Dr. H. W. McConnel is making inquiries by circular at other sanatoria in order to find out if there is really any foundation for the rumours of hardship in this respect. Dr. McConnel is the Honorary Secretary of Kelling Sanatorium, and is presumably bearing the expense of this inquiry himself. He asks a series of pertinent questions, among which is the following: "Would it be likely to make the ex-soldier more contented at the sanatorium if he had a period of furlough before he commenced the treatment?" It is doubtful if very much reliable information will be obtained from the replies to this circular, for some of the questions are difficult, and a good deal of retrospective research would be necessary adequately to answer them. To the last question, "Would it be desirable to attempt to obtain concerted action in the matter of assisting tuberculous soldiers to get adequate pensions?" we fancy a good many medical superintendents will reply to the effect that the politicians have more time and more taste for that sort of agitation.

### THE LATE SIR CHARLES BALL.

The medical profession and the institutional world in Ireland lose one of their leading personalities by the death of Sir Charles Bent Ball, Bart., F.R.C.S., and temporary lieutenant-colonel in the Royal Army Medical Corps. He was born in 1851, and was a younger brother of Sir Robert Ball, the astronomer. He graduated in medicine at Trinity College, Dublin, in 1872, and achieved a great name as an all-round surgeon with an especial reputation for his skill in the surgery of rectal diseases. Sir Charles was honorary surgeon to the King in Ireland; Lord Chancellor's Consulting Surgical Visitor in Lunacy; consulting surgeon to Steeven's Hospital and to other Dublin institutions; surgeon to Sir Patrick Dun's Hospital and to Simpson's Hospital, Dublin; Regius Professor of Surgery at Dublin University; Member of the Council of the Irish Royal College of Surgeons; representative of Dublin University on the General Medical Council; late Member of the Advisory Board for the Army Medical Service. He was knighted in 1903 and created a baronet in 1911; in the latter title he is succeeded by his eldest son, Dr. C. A. K. Ball, who, like his late father, is surgeon to Sir Patrick Dun's Hospital, and has made a special study of diseases of the urinary tract.

### HOSPITAL ARRANGEMENTS IN MESOPOTAMIA.

The growing impression among the public that hospital arrangements for our forces in Mesopotamia have been badly mismanaged has found expression lately, both in the Houses of Parliament and in the Press. Officially there is very little information to be got, but unofficially it is apparently established that a very gross failure to foresee the requirements of the situation has been exhibited. An official statement appeared in the *Times* on March 15, which showed that much activity is now going on in various circles to supply hospital necessaries. The Joint Committee of the Order of St. John and of the British Red Cross Society is evidently working heroically to make good numerous and great deficiencies in requisites. But reading between the lines of this document it is impossible not to conclude that the authorities have only just realised, through popular indignation, what with their vastly superior sources of information they ought to have been well aware of months ago. Now, evidently, they admit that the medical service in Mesopotamia has been starved, and are turning eagerly to anyone who will help them out of their difficulties. What the public wants, and justly, is to fix the responsibility for a state of things which is not only disgraceful in itself, but utterly inexcusable when the shining example of the medical arrangements for our forces in France exists for emulation.

### GUY'S HOSPITAL.

We are favoured by an advance copy of some of the figures of the accounts of Guy's Hospital for 1915 contained in a statement showing the comparison with the previous year. There was an excess of expenditure over income for the year of £4,801, as against an excess of income over expenditure in 1914 of £27,860. The difference arises out of the facts that in the earlier year £22,107 more was received from legacies, while in 1915 the ordinary expenditure increased by £6,070. In respect of ordinary income, although annual subscriptions fell off by nearly 3 per cent., and the King's Fund grant was £500 less, the totals were £71,308, as against £70,182. Under the chief maintenance headings of expenditure there was an increased cost of from 3.78 per cent. ("Salaries, wages, etc.") to 22.59 per cent. ("Domestic"), with the exception of that of "Surgery and dispensary," under which the expenditure fell from £8,795 to £7,129, or nearly 19 per cent. In reference to the last result it is most probable that the stocks of drugs, etc., on January 1, 1915, were appreciably higher than at the end of the year. In hospital accountancy at the present time no estimates are published as to the values at the end of the financial year of stocks of linen, drugs, dressings, etc., and where such a remarkable and unexpected result as that quoted presents itself some qualifying note might usefully be supplied. No doubt the report to the governors will contain the necessary information. The cost of provisions rose from £17,019 in 1914 to £20,677 in 1915, or by 21 per cent.

### DOCTORS REPROVED BY INSURANCE COMMITTEE.

In the county of Radnorshire there were in one quarter last year fourteen deaths from tuberculosis, but during that period only four cases were notified to the county medical officer. Attention was called to this statement at a meeting of the Insurance Committee, and the chairman agreed that it was a serious matter. If things went on like that, he said, the work of the Memorial Association would be reduced to a farce. On the face of it there seems to have been default on the part of the local medical practitioners to notify, but it was suggested that part of the explanation was that some of the cases were dealt with by the military authorities. Possibly the friends of the patients were reluctant to report cases until too late—a contingency not at all unfamiliar to tuberculosis officers in Wales. In any case it is hardly conceivable that the doctors of Radnorshire deserve the strictures passed upon them. It is pure exaggeration to describe them as deliberately "setting at naught the efforts of those who were trying to stamp out the white plague."

### THE HOSPITAL LESSON OF BRISTOL.

We lately alluded to the piece of great good fortune in the shape of the Capern legacy of £45,000 which has crowned with success the financial expedients of the Bristol Royal Infirmary. The Bristol General Hospital has been distinguished by a cautious policy, which has been jealous of realising securities and gone quietly and calmly on, alive to the need for developments, but chary of embarking on new schemes till ways and means have been provided. The General Hospital has also, as we noted, received £45,000 from the Capern bequest, and so each institution has succeeded by different methods suited to its character and growth. To contrast the developments at the General Hospital with the great new Memorial Infirmary is the better to understand both institutions. Mr. Fenwick Richards has given a new chapel, whereby the old has been converted into a ward; the office of Mr. T. Gregg, the secretary, has been enlarged; an internal telephone and a water-softening plant have been installed. Then the new wing, for which they had hoped to have provided an endowment fund, in accordance with the practice of not rushing unduly into debt, has been endowed by the £2,000 a year to be provided from the interest of the Capern legacy. Progress is a matter of results; it is not the quality of a method. The development of the two great hospitals at Bristol is a classic case in point.

### AN IRISH PHILANTHROPIST.

With the death of Mr. James Davidson, at the age of eighty, the Royal Victoria Hospital, Belfast, has lost a firm friend and former chairman of the board of management. Mr. Davidson, who made his mark as a man of business in Assam, where he was a pioneer tea-planter, was typical of that class of Ulsterman who, after gaining success in business, turned his attention and enterprise to hospital work. He was very proud of his institution, and the King Edward Memorial extension, which was opened by the Lord-Lieutenant in May last year, owed a great deal alike to his generosity and to his energy. In the days of the Frederick Street Hospital he acted as honorary treasurer, but this official work was not the end of his interest. He was a constant visitor to the wards, and made himself familiar with the working of every department. The extension, which comprises, as our readers may recall, a vaccine therapy and hæmatological department, together with a department for medico-electrical therapeutics, was his pride and care, and his interest in detail was also constant. The hospital's two conspicuous clocks, in the façade and in the entrance hall, were his gifts, and the staff, as well as the management, have lost a well-tried friend in him.

### AMUSEMENT AS THE BASIS OF ORDERLY CONDUCT.

Through the accounts which we have lately been able to give of the *Ghain Tuffieha Hospital Record*, Malta, our readers will be able to appreciate the value of the tribute paid to hospital organisation in the island by the Governor, Field-Marshal Lord Methuen, in a minute issued by him to the Director of Medical Services. His Excellency records that 60,300 patients have received treatment in the Malta hospitals since May 1915. The number of beds, which had been raised from a few hundred to 20,000, could now accommodate in one month 25,000 patients. But what will interest our readers most is Lord Methuen's praise of the orderly conduct of the patients, which he attributes to the admirably organised concerts and amusements in the hospital camps. In our review of the canvas camp journal referred to above, we noted particularly the liveliness of the camp life indicated in its pages from the accounts of sports, concerts, debating societies, and other forms of recreation which, with articles on more serious topics, helped to give a vivid picture of the life and activity of the camp. Colonel Maurice, C.M.G., R.A.M.C., the editor of the paper and the officer in command of the camp, will share with his colleagues in the satisfaction created by this official recognition of their efforts on the patients' behalf.

### A DESPERATE EXPEDIENT AT YORK.

For a long time past the authorities of the York County Hospital have been complaining of financial anxieties, and have been seeking about for one expedient or another to stave off an indebtedness which would cause disaster. The latest move in this direction is a change in the rules, whereby all legacies which are not specially earmarked for investment may be used for general purposes. After much discussion it was decided last year to issue a special appeal, which had a small measure of success, and the financial year has ended with an excess of income over expenditure, and a reduction of the adverse balance from £3,880 to £2,554. The workpeople's hospital fund reached £1,813, or £400 more than in 1914. Notwithstanding the lesson of this improvement, which is, as we have reiterated many times, that success is the fruit of a vigorous policy and is not to be purchased by this or that device—the dangerous expedient of placing all legacies to the current account has been adopted. The extent to which legacies should be invested is a legitimate subject of debate; but we deplore the decision at York to spend them as ordinary income because it seems to show that the policy of drift and expedients is not yet done with. We believe that greater effort would have made last year's appeal far more successful than it was. We also believe that the promise of the workpeople's fund can be made to bear rich fruit, and that other funds for other classes can and should be organised. Attempts on these lines would indicate a constructive policy. The decision to appropriate legacies is only a shift of desperation.

### ORDINARY COMPETENCE AND SKILL.

In the agreements which are entered into between panel practitioners and Insurance Committees there is a clause which provides that the practitioner shall give to all his patients treatment of a kind "which can consistently with the best interests of the patients be properly undertaken by a general practitioner of ordinary competence and skill." Obviously opinions may easily differ as to what constitutes ordinary competence; and panel practitioners are under some temptation to belittle it when they see a chance of extra remuneration for some particular professional service. In illustration of the point, two cases dealt with at the March meeting of the Panel Committee of the County of London suffice well enough. A panel practitioner made a charge to a panel patient for doing retinoscopy under atropine, and for prescribing glasses as a result of it. The patient complained of this charge, contending that the service should be rendered free under the panel agreement. It is notorious that only about one general practitioner in a hundred can do refraction work at all, and we agree with the Panel Sub-committee that (as matters stand) the doctor was justified in making a charge. But this state of things should not be. The Medical Council should see to it that medical qualification entails much more knowledge of ophthalmic surgery than in fact it now does, and retinoscopy should not be, as it admittedly is, beyond the competence of the average practitioner.

### PROPHYLAXIS NO TREATMENT.

The other matter concerned vaccination, a procedure which the Panel Sub-committee regarded as not constituting treatment within the meaning of the panel agreement. Whether this is or is not a correct view it may take lawyers and juries to decide; but from the medical, as opposed to the legal, aspect we should have thought that vaccination against smallpox, or inoculation against enteric, was just as much "treatment" as, let us say, the anti-tetanus serum treatment of a badly lacerated and deeply contaminated wound. Surely the whole trend of medical progress and medical education of the present day is more and more towards prevention as the most rational and economical form of cure; and, although the exact interpretation of a clause in a panel agreement will not seriously impair the position of medical science in general towards the community at large, it does seem to us undesirable that a body of medical men gathered together in council should attempt to restrict the meaning of the word treatment so as to exclude prophylaxis from its scope.

### THE END OF AN OLD DISPENSARY.

Competition is not always illegitimate even in the sphere of charitable work, and the closing of Norwich Dispensary this month, after some 112 years of usefulness, has been apparently due to this cause. About 1890, what proved a fatal step was taken when the late Mr. John Gurney helped to establish a provident department. Indeed, by 1892 £809 were subscribed by the provident members. This was the beginning of the end. Charitable contributions decreased as those of the provident members waxed; and the sincerest form of flattery, in the cant phrase, was paid to the provident department when, its success being apparent, a "Public Medical Service" was started on similar lines in the town. The lines were not identical, however, for while the dispensary paid over to the medical staff, less 15 per cent. for standing charges, all the subscriptions of the provident members, in the case of the "Public Medical Service" all expenses, we understand, are paid by the latter body without the external help (such as the charitable funds at the dispensary's disposal afforded). As the "Public Medical Service" is popular among local practitioners, who have doubtless evolved a plan agreeable to themselves, and the scheme is self-supporting, the trustees of the dispensary have decided to close it, and their feelings on the matter were endorsed at a recent meeting of the subscribers. Its invested funds of some £2,500 and some real property remain to be disposed of, and the trustees are considering which other charity should benefit by them when the subscribers will again meet to sanction the deed of gift which will be the last public benefit to be conferred by a career of usefulness which has lasted over 100 years.

### THE TEST OF GENERAL ABILITY.

The congratulations of their professional colleagues were given last week to Mr. Edgar A. Browne, ophthalmologist, and Dr. George C. Walker, of Southport, alienist, on the completion of fifty years' membership of the Liverpool Medical Institution. Major Dr. Macalister, who presided, referred to the professional careers of both members, and after allusion by more than one speaker had been made to Mr. Browne's powers of repartee and interest in literature, a resolution setting forth the two members' professional record was passed congratulating them on their medical jubilee. In connection with Mr. Browne's professional repute and reputation as a wit and amateur of letters, we may recall an aphorism made to us by a distinguished medical writer. On hearing someone say that the literary faculty was not nearly so uncommon as it is generally supposed to be (at least by authors!) our friend replied: "That is hardly the way to put it, what I do not think is recognised enough is the rareness with which any marked ability is unaccompanied by the literary faculty." Would not his friends quote Mr. Edgar Browne as a case in point?

### THE ASYLUM AND SIGNS OF CHANGE.

Dr. George M. Robertson, physician superintendent of the Royal Edinburgh Asylum, rarely loses an opportunity of driving in a little further the thin edge of the wedge of reform which, with occasional set-backs, has divided the modern mental hospital by a growing gulf from the nineteenth century asylum. In dealing with the signs of change which are beneficently apparent in mental institutions, he has not only emphasised the reform brought about by the introduction of female nurses, under suitable conditions, in the male wards, but he has also pointed to the approaching revision of the unsatisfactory Lunacy Laws which still clog progressive administration. Now that the treatment of insane soldiers has been provided for in homes and institutions without the stigma of certification, it is clear that the new methods cannot subsist for ever with the old, and that the old is sufficiently entrenched to oust the new is unthinkable. Dr. Robertson remarks that a cloud of witnesses, drawn from all who have had to work under the Lunacy Laws, could testify against them when the end of the war provides an opportunity of doing so with effect—before the last soldier patient has been disposed of. When, in addition to the above changes, the status of the assistant medical officers is reconsidered, and the virtual bar to marriage and promotion (often even to interesting work) removed, the era of the twentieth century mental hospital will have begun in earnest.

### CROYDON HOSPITAL WITHOUT RESIDENT DOCTOR.

Croydon General Hospital is without a resident surgeon—a fact which caused Dr. T. Jackson, the Borough Coroner, to offer strong comments on the fact that a hospital with over a hundred beds should be in such a position. While holding an inquest he pointed out that a man taken to the hospital shortly after midnight was not seen by a medical man until eight hours or more afterwards. Dr. Davidson, a well-known local practitioner, who attended the man in question, pointed out that he had been attended to by a gentleman who had had considerable e perience, and only had to pass one more examination before he was qualified. He had been too busy since the war began to study. Dr. Jackson, however, retorted that the man was not qualified, and something ought to be done. He also stated that in the advertisements it was not stated that a salary would be paid. He hoped that as the result of his remarks something would be done.

### THE LONDON MENTAL HOSPITALS COMMITTEE.

The London County Council has recently appointed the Mental Hospitals and Mental Deficiency Committee which is to serve during the coming financial year. Full use has been made of the power of co-option now conferred upon the Council, for among the outside members of the committee we notice the name of Sir John McDougall, who did sterling work for the mental hospital service for many years when he was a member of the Council, and who is now a member of the sub-committees which control seven of the mental hospitals. Among the lady members are Mrs. Herbert Curteis, Mrs. Dunn-Gardner, Miss M. H. Joseph, Miss Evelyn Fox, Miss E. M. Henniker, Miss Ida Samuel, Mrs. Wilton Phipps, and Lady St. Helier, who, of course, is also the energetic almoner of the County of London War Hospital at Horton. Mr. Cyril Cobb and Mr. H. V. Rowe have been reappointed chairman and vice-chairman respectively of the committee.

### THE "VIS INERTIA" IN A SCOTCH ASYLUM.

The controversy which has arisen over the resignation of Mr. Birrell, convener of the Dundee Asylum, is instructive rather from the suggestions that it has excited than for the matter at issue. Mr. Birrell resigned on the ground that his colleagues failed to give him support. Those of his colleagues, however, who are not his supporters, while admitting his energy and disinterestedness, excuse themselves by stating that he was expecting too much too quickly, and that, significant point, his real difficulty has been not his colleagues' faint-heartedness, but "the heavy *inertia* of the settled condition of things. No doubt *inertia* is as necessary to change and movement as friction is to the wheels on the road, only while we cannot have friction without movement in traction, friction without movement is possible enough in Parliamentary and institutional affairs. The *vis inertia* is active *inertia*; the deliberate putting on of the brake. The art of administration consists in preventing the brake from jamming so that the wheels will sink into the mud of "the settled condition of things." This is what has happened in the House, which should be a monumental warning to the humblest house committee.

### THE NEW ABERAVON HOSPITAL.

A new general hospital has been built and opened at Aberavon. The site, which extends to nearly two acres, has been given by Sir A. Pendarves Vivian, K.C.B., and the building itself has cost £4,000. The chairman of the General Committee is Mr. S. H. Byass, and the employees of Messrs. R. B. Byass and Co., Mansel Tin Works, have decided to raise subscriptions for a receiving ward, to be built and named in memory of Lieut. Rupert Hallowes, V.C., formerly assistant manager at the works, who was killed in France last September. The hospital provides for fourteen beds and two cots, but eventually it is hoped to complete the building by certain foreseen additions which will bring up the total number of beds to fifty. Financially, the outlook is promising, since the building fund is only £500 below the £4,000 required, and Mr. Charles Routledge, chairman of the house committee, was able to announce at the opening that while the estimated cost of maintenance was £1,200, a sum of £850 had been promised or obtained. No more satisfactory send-off could be imagined.

### A CURIOUS COTTAGE HOSPITAL PRACTICE.

Cottage hospital management is admittedly more informal than can be the case in the larger institutions, but few cottage hospitals have made a practice of combining the offices of president and (active) hon. secretary. This, however, has been the case at the Braintree and Bocking Cottage Hospital for sixteen years, during which Mr. W. J. Courtauld has held the two offices. He has lately expressed the opinion that the two offices should be filled by two different people. He is certainly right in this; for the official head of an institution should not be one of the workaday staff. This, the simplest of cottage hospitals, is one everybody should visit. A description and photograph of it were published in The Hospital of November 22, 1913, page 198.

### WHY DO SANATORIUM PATIENTS COMPLAIN?

The investigation of complaints made by sanatorium patients is so generally followed by an apparent absence of any reasonable cause that thoughtful persons may well consider why complaints recur. It is needless to add that their recurrence but rarely reaches the newspapers. Why do they persist, however? Ask the experienced sanatorium medical officer (not quite the rare bird he was ten years ago), and he will tell you that the superintendents appointed under the George scheme had had no experience in the special art of managing sanatorium patients. Ask the young assistant medical officer, and he would probably reply that the patients were naturally undisciplined, and had been made more intractable by the cant of the politicians who led them to expect "first-class hotels"—anything less suited to consumptive patients can hardly be imagined! Ask the patients the cause of their restiveness, and they would complain of "being at school," of the doctors, and of the regulations.

### THE HEEL OF ACHILLES IN SANATORIUM TREATMENT.

Of these answers the experienced medical officer's criticism of the appointments made, and the patients' criticisms of the rules and doctors, come nearest the truth. But from the medical superintendent's point of view we fancy that the truth should be this: while tact on the part of the staff is the more necessary as the regulations are the more precise, the fact is that sanatorium treatment is so largely a matter of psychology on the part of the patient that psychological considerations in selecting suitable cases for treatment are just as important as the physical and economical facts to which alone regard is paid at present. The omission to pay heed to temperamental as well as to physical signs in the patient is the heel of Achilles in sanatorium work. As it is, a patient and a doctor have even been known to come to blows!

### A CORPORATION CARE COMMITTEE.

The care of consumptives in the Borough of Wigan has been for two years entrusted to an admirably conscientious committee, whose work on the usual "after-care" lines was freely praised by leading townsmen at the recent annual meeting. During the year 106 poor consumptives were helped in various ways according to the needs of each case. Besides gifts of milk and other food and clothing, bedding and bedsteads have been lent to patients who otherwise would have been unable to sleep alone. Both insured and non-insured persons were assisted, and it is hoped that the projected extension of the committee's work among children and the provision of residential treatment will not be long deferred. The question of starting an open-air school is to be considered by the Education Committee. The financial report, it is gratifying to learn, is quite satisfactory, and shows a balance on the right side. At this meeting the Town Clerk announced that in future the after-care of consumptives would be put under the official control of the Corporation. This meant that some changes would be henceforth necessary in regard to the election of members of the Care Committee. Nominations should be forwarded to the Corporation, and on these that body would appoint the members in future. Meanwhile the Care Committee was re-elected until a proper scheme had been approved by the Local Government Board.

### AN EDUCATIONAL CAMPAIGN TO COMBAT VENEREAL DISEASE.

No time is to be lost by the National Council for Combating Venereal Diseases in making a beginning of an educational campaign which it is proposed to organise. The executive committee of the Council are appealing for financial assistance towards the cost of arranging conferences and courses of lectures. The principal points in the report of the Commission are to be summarised and embodied in a suitable pamphlet for immediate distribution. The Hospital has in all references to this subject insisted upon the absolute necessity of increased facilities for treatment of these diseases as a step of primary importance in lessening the evil due to venereal affections. Certainly little can be done in the way of compulsion until special treatment is readily available. It is to be hoped that the National Council will employ some of their effort in this direction.

### THIS WEEK'S DRUG MARKET.

The drug market continues fairly active, and on the whole prices are well maintained, with here and there sharp advances in value. Owing to the difficulty of getting shipments the scarcity of citric and tartaric acids has become very pronounced, and prices have advanced accordingly. At the moment there is practically no difference between the price of citric acid and tartaric acid, a most unusual condition, as tartaric acid is commonly regarded as a cheap substitute for citric acid. Both these acids are largely used at this season of the year in the manufacture of effervescent saline preparations, the cost of which has risen substantially. Cream of tartar is slightly dearer. Iron and ammonium citrate, sodium citrate, and potassium citrate are all dearer, and makers of tartrates will no doubt raise their prices. The very high prices quoted for cod-liver oil continue to keep buyers off the market, and as the fishing conditions improve it is not improbable that dealers will have to become less firm in their views. No changes of note have taken place in the prices of synthetic drugs. Gentian root is again dearer. The scarcity of belladonna root is unrelieved. A good business has been done in gum acacia, and prices have an upward tendency. Castor oil, which has been steadily advancing in price for a long time, now shows signs of receding in value slightly. A good business has been done in menthol at full prices. There seems to be little reason to anticipate any fall in makers' prices for bromides, notwithstanding that the demand continues quiet for the time being.

### TO OUR READERS.

**Contributions are specially invited from any of our readers to these columns. They should deal with topical subjects and news. They must be authenticated for the information of the Editor only. The minimum payment if published is 5s. There is no hard-and-fast rule as to space, but notes of about twenty lines in length are preferred.**

